# Spherical nucleic acid targeting microRNA-99b enhances intestinal MFG-E8 gene expression and restores enterocyte migration in lipopolysaccharide-induced septic mice

**DOI:** 10.1038/srep31687

**Published:** 2016-08-19

**Authors:** Xiao Wang, Liangliang Hao, Heng-Fu Bu, Alexander W. Scott, Ke Tian, Fangyi Liu, Isabelle G. De Plaen, Yulan Liu, Chad A. Mirkin, Xiao-Di Tan

**Affiliations:** 1 Center for Intestinal and Liver Inflammation Research, Stanley Manne Children’s Research Institute, Ann & Robert H. Lurie Children’s Hospital of Chicago, Chicago, IL 60611, USA; 2 Department of Pediatrics, Feinberg School of Medicine, Northwestern University, Chicago, IL 60611, USA; 3 Department of Chemistry and the International Institute for Nanotechnology, Northwestern University, Evanston, IL 60208, USA; 4 Department of Biomedical Engineering, Northwestern University, Evanston, IL 60208, USA; 5 Department of Gastroenterology, Peking University People’s Hospital, Beijing 100044, P. R. China; 6 Department of Pathology, Feinberg School of Medicine, Northwestern University, Chicago, IL 60611, USA

## Abstract

Milk fat globule-EGF factor 8 (MFG-E8) maintains the intestinal homeostasis by enhancing enterocyte migration and attenuating inflammation. We previously reported that sepsis is associated with down-regulation of intestinal MFG-E8 and impairment of enterocyte migration. Here, we showed that impairment of intestinal epithelial cell migration occurred in lipopolysaccharide (LPS)-induced septic mice. Treatment of RAW264.7 cells (a murine macrophage-like cell line) with LPS increased expression of miR-99b, a microRNA that is predicted to target mouse MFG-E8 3′UTR. Using a luciferase assay, we showed that miR-99b mimic suppressed the activity of a reporter containing MFG-E8 3′UTR. This suggests the role of miR-99b in inhibition of MFG-E8 gene expression. In addition, we developed an anti-miR99b spherical nucleic acid nanoparticle conjugate (SNA-NC^anti-miR99b^). Treatment of both naïve and LPS-challenged cells with SNA-NC^anti-miR99b^ enhanced MFG-E8 expression in the cells. Administration of SNA-NC^anti-miR99b^ rescued intestinal MFG-E8 expression in LPS-induced septic mice and attenuated LPS inhibitory effects on intestinal epithelial cell migration along the crypt-villus axis. Collectively, our study suggests that LPS represses MFG-E8 expression and disrupts enterocyte migration via a miR-99b dependent mechanism. Furthermore, this work shows that SNA-NC^anti-miR99b^ is a novel nanoparticle-conjugate capable of rescuing MFG-E8 gene expression and maintaining intestinal epithelial homeostasis in sepsis.

Epithelial cell migration plays an important role in maintaining intestinal epithelial homeostasis. Under the normal physiological state, epithelial cells migrate up along the intestinal crypt-villus axis, which contributes to renewal of the epithelial lining every 3–5 days^1^,^2^,^3^,^4^. During mucosal wound healing, restitution (a process of migration of intestinal epithelial cells adjacent to the injured surface into the wound) is a critical event for resealing mucosal damage^5^. Previous studies have shown that the migration of intestinal epithelial cells is impaired in numerous critical illnesses. For instance, we have revealed that sepsis is associated with delayed intestinal epithelial cell migration along the crypt-villus axis^6^. Evidence shows that inflammatory mediators including nitric oxide and extracellular high mobility group box-1 inhibit intestinal epithelial restitution^7^,^8^. Disruption of intestinal epithelial restitution results in delaying mucosal wound healing. Currently, effective approach for maintaining intestinal cell migration in critical illnesses is lacking.

MFG-E8 is a secreted protein^9^,^10^. Originally, MFG-E8 was found to bind both α_v_β_3_ integrin and phosphatidylserine, which facilitates clearance of apoptotic cells by macrophages^11^. Recently, MFG-E8 has been shown to mediate multiple physiological and pathophysiological events besides the removal of apoptotic cells. For example, it has been reported that MFG-E8 contributes to attenuating neutrophil infiltration in lungs via modulation of CXCR2^12^. Evidence further shows that MFG-E8 inhibits tissue fibrosis by promoting the removal of collagens from inflammatory tissues^13^. Collectively, it appears that MFG-E8 has diverse biological functions.

In the gut, MFG-E8 is produced by lamina propria macrophages^6^. We previously showed that MFG-E8 promotes intestinal epithelial cell migration *in vitro* and *in vivo*^6^. Furthermore, we and others demonstrated that MFG-E8 is a critical protein for attenuating colitis in mice and humans^14^,^15^,^16^. In addition, Ajakaiye *et al*. reported that recombinant MFG-E8 protects against mucositis induced by whole body irradiation^17^. Taken together, MFG-E8 is suggested to be an important protective factor for gut epithelial homeostasis.

Intestinal MFG-E8 expression is suppressed in inflammation^6^,^15^. Down-regulation of MFG-E8 contributes to delayed intestinal epithelial cell migration and restitution in inflammation^6^,^15^. However, our knowledge about the molecular mechanisms underlying suppression of MFG-E8 gene expression in inflammation remains incomplete. Previously, we have studied the 5′-promoter region-associated transcriptional regulation of the MFG-E8 gene expression in physiological and inflammation conditions^18^. It is well known that not only 5′-promoter region but also the relationship between microRNAs (miRNA) and their binding motifs in 3′UTR of mRNAs influence gene expression. Therefore, in the present study, we further examined the involvement of 3′UTR and miRNAs in suppressing MFG-E8 gene expression in lipopolysaccharide (LPS)-induced sepsis condition. In addition, to protect against sepsis-induced impairment of intestinal homeostasis, we developed a novel approach which specifically targets MFG-E8 associated miRNA, thus rescuing MFG-E8 gene expression, using spherical nucleic acid (SNA) nanoparticle conjugate (NC) technology.

## Results

### LPS inhibits enterocyte migration along the crypt-villus axis

**Figure 1:**
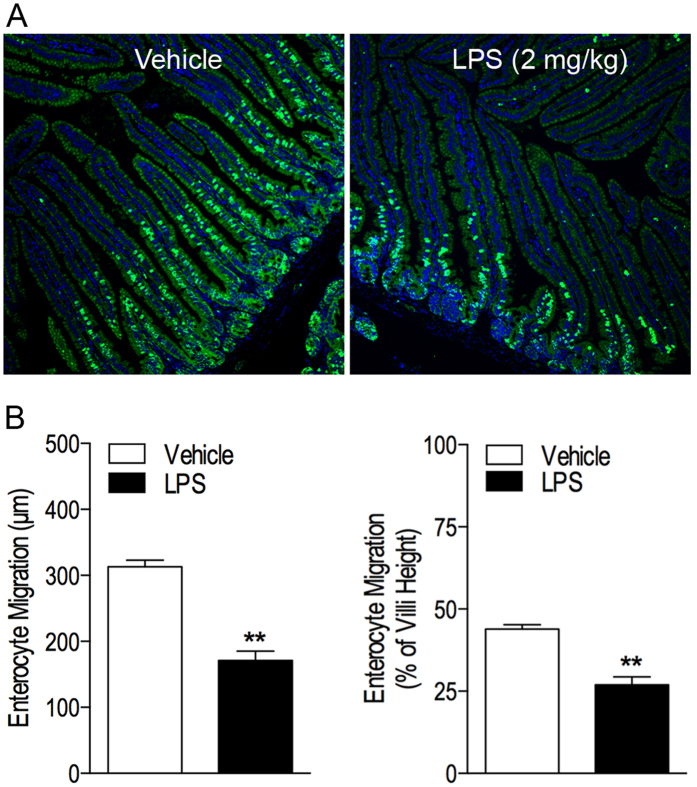
LPS inhibits enterocytes migration *in vivo*. Mice (C57BL/6J, male, 7 weeks old) were treated with LPS (2 mg/kg) or vehicle (saline, equivalent volume per mouse). After 24 h, mice were given BrdU (50 mg/kg, i.p.) for pulse-chase labeling intestinal epithelial cells to study their migration along the crypt-villus axis. They were sacrificed at 48 h after BrdU administration. Small intestines were harvested and processed for routine histology. Tissue sections were immunofluorescently stained with anti-BrdU antibody. Nuclei were counterstained with 4′,6-diamidino-2-phenylindole (DAPI). The migration of BrdU-labeled enterocytes along the crypt-villus axis was analyzed as described in Methods. **(A)** Typical fluorescent microscopy images of BrdU/DAPI-stained small intestinal tissues in each treatment group. Merged images. BrdU-labeled cells are colored green. Nuclei are colored blue. 10X. **(B)** Quantitative analysis of BrdU-labeled enterocyte migration by measuring the distance from the base of the crypts to the highest labeled cell within the crypt-villus axis (left panel) and the migration distance as a percentage of total villus height (right panel) in the jejunum. n = 5. **P < 0.01 versus vehicle group.

Sepsis is associated with endotoxemia that is likely to contribute to the pathophysiological features of sepsis. To understand the role of endotoxemia in intestinal epithelial barrier homeostasis, we studied the pathophysiological effect of LPS-induced sepsis on enterocyte migration along crypt-villus axis in mice using *in vivo* bromodeoxyuridine (BrdU) pulse-chase analysis. First, we found that intestinal epithelial cells were labeled with BrdU *in vivo* (Fig. 1). The labeled enterocytes were migrated along the crypt-villus axis, suggesting turnover of the intestinal epithelium under normal physiological conditions. Furthermore, we demonstrated that intraperitoneal inoculation of LPS (2 mg/kg) resulted in impairment of enterocyte migration along the crypt-villus axis (Fig. 1A). Quantitative analysis of the distance that BrdU-labeled cells had migrated from the crypt further showed that enterocyte migration along the crypt-villus axis was reduced 45.3% (i.e. from 313 ± 9.98 to 171 ± 14.31 μm, *P* < 0.01) at 48 h post-BrdU labeling (Fig. 1B) in LPS-treated animals, compared with vehicle treatment. In addition, the BrdU-labeled enterocytes were found to replace 43.9% ± 1.35 of intestinal epithelial cells in villi in vehicle-treated mice (Fig. 1C). In contrast, the rate of intestinal epithelial cell turnover was reduced to 26.9% ± 2.49 at 48 h post-BrdU labeling in LPS-treated animals, corresponding to a 38.7% decrease compared with vehicle treatment (*P* < 0.01). Together, the data suggest that LPS-induced systemic inflammation is associated with reduction of intestinal epithelial cell migration along the crypt-villus axis.

### LPS induces miR-99b expression in RAW264.7 cells

**Figure 2:**
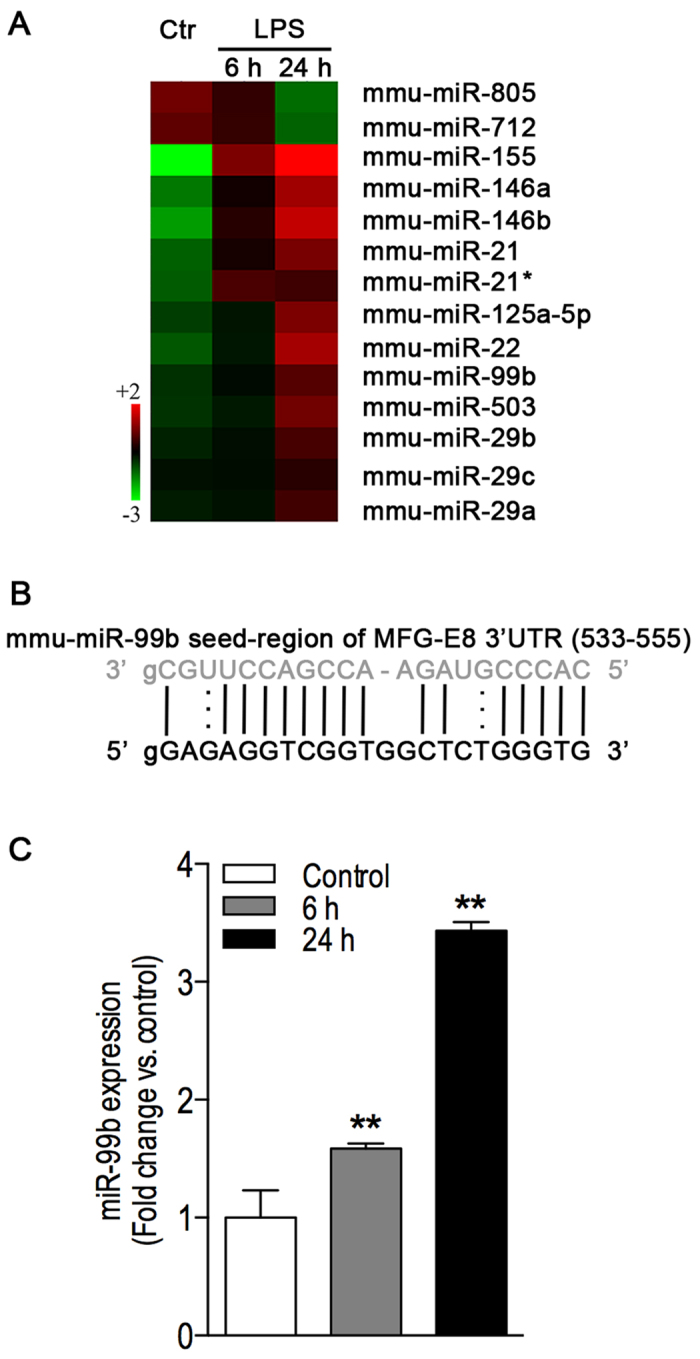
LPS induces expression of miR-99b that potentially targets on MFG-E8 3′UTR. **(A)** Heatmap of miRNA expression microarray showing dominant differentially regulated miRNAs in LPS-challenged macrophages. RAW264.7 cells were treated with LPS (100 ng/ml). The control cells were treated with culture medium alone. The colors of the heatmap are normalized expression values. Green represents relatively low expression, red relatively high expression, and black relatively intermediate expression within the scope of each miRNA probe. **(B)** Predicted the miR-99b target site in the 3′UTR of the mouse MFG-E8 mRNA. **(C)** RT-qPCR validation of miR-99b expression in LPS-challenged RAW264.7 cells. The cells were treated with LPS (100 ng/ml) for 6 h and 24 h respectively. The control cells were treated with culture medium alone. Total RNA including microRNA was extracted. miR-99b levels were evaluated with TaqMan RT-qPCR microRNA assay. The expression of miR-99b was normalized relative to the expression of Sno202 (a housekeeping miRNA). Values are mean ± SEM and represent average of findings from two independent experiments with triplicate samples in each group. **P < 0.01 versus the control group.

MFG-E8 gene expression is regulated transcriptionally and post-transcriptionally. Recently, we elucidated a mechanism underlying transcriptional regulation of MFG-E8 gene expression by LPS^18^. However, little is known about post-transcriptional regulation of this gene in inflammation. miRNAs are a class of small non-coding RNAs with around 22 nucleotides in length. A body of evidence has shown that miRNAs repress gene expression at the post-transcriptional level via targeting 3′UTR regions^19^,^20^. Given the length of the MFG-E8 3′UTR (641 nucleotides), we utilized a database available at http://microrna.sanger.ac.uk/targets/v5/ to identify potential miRNA targets on murine MFG-E8 mRNA 3′UTR. The bioinformatic approach revealed presence of 48 microRNA-binding sites in mouse MFG-E8 3′UTR. To further identify candidate miRNAs involved in the LPS action, we performed a miRNA array of LPS-treated RAW264.7 cells, a murine macrophage-like cell line. As shown in Fig. 2A, LPS induced expression of a sizable subset of miRNAs such as miR-21, miR-22, miR-29 family, miR-99b, miR-146a-b, and miR-155 in RAW264.7 cells. Furthermore, analysis *in silico* revealed that the MFG-E8 3′UTR comprises a putative consensus sequence for mmu-miR-99b (Fig. 2B). Thus, real-time RT-qPCR was carried out for verifying expression of miR-99b in LPS-treated RAW264.7 cells. We found that LPS significantly induced expression of miR-99b in a time-dependent manner (Fig. 2C).

### miR-99b targets the 3′UTR of murine MFG-E8 mRNA

**Figure 3:**
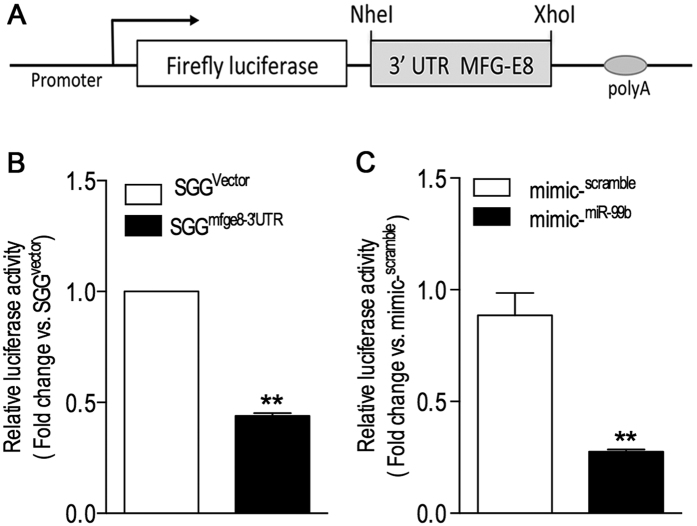
miR-99b directly targets MFG-E8 3′UTR. **(A)** The schematic diagram of pSGG^mfge8-3′UTR^, a luciferase reporter construct containing murine MFG-E8 3′UTR. **(B)** MFG-E8 3′UTR is a functional unit for maintaining homeostasis of MFG-E8 mRNA. RAW264.7 cell were transiently transfected with pSGG^mfge8-3′UTR^ and pSGG^vector^ (i.e. control) respectively. After 48 h, cell lysates were processed for luciferase assay as described in Methods. Values are mean ± SEM and represent average of findings from two independent experiments with triplicate samples in each group. **P < 0.01 versus the control group. **(C)** miRNA-99b mimic inhibits MFG-E8 3′UTR activity. RAW264.7 cells were transiently co-transfected with pSGG^mfge8-3′UTR^ and mirVana^TM^ miR-99b mimic. As a control, the cells were co-transfected with pSGG^mfge8-3′UTR^ and mirVana^TM^ mimic scramble instead. After 48 h, cell lysates were processed for luciferase assay as described in Methods. Values are mean ± SEM and represent average of findings from two independent experiments with triplicate samples in each group. **P < 0.01 versus the control group.

To further determine the exact role miR-99b in the regulation of the MFG-E8 3′UTR function, we sub-cloned a 641-bp segment of murine MFG-E8 3′UTR into the pSGG luciferase miRNA target expression vector to generate a reporter named pSGG^mfge8-3′UTR^ (Fig. 3A). Then, the function of murine MFG-E8 3′UTR and the effect of miR-99b on regulation of MFG-E8 translation activity were assessed through transfection of pSGG^mfge8-3′UTR^ into RAW264.7 cells and luciferase activity assay. We found that the level of luciferase activity in the cells transfected with pSGG^mfge8-3′UTR^ were significant lower than that in cells transfected with empty vector (Fig. 3B), suggesting that the MFG-E8 3′UTR is a functional unit for maintaining homeostasis of MFG-E8 gene expression at a post-transcriptional level. Furthermore, treatment with miR-99b mimic significantly decreased reporter activity of pSGG^mfge8-3′UTR^ (Fig. 3C), suggesting that miR-99b inhibits MFG-E8 posttranscriptionally by targeting to its 3′UTR.

### miR-99b mediates the effect of LPS on repression of MFG-E8 expression *in vitro* and *in vivo*

**Figure 4:**
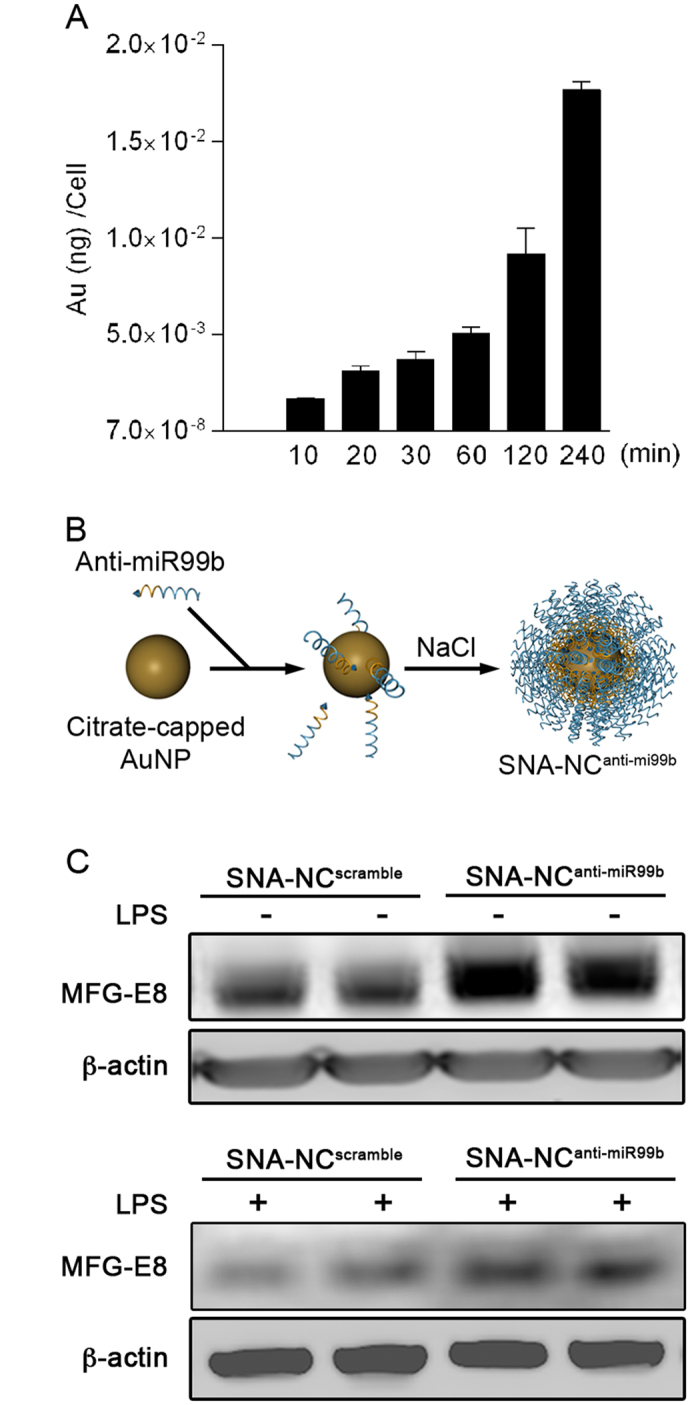
SNA-mediated delivery of miRNA-99b inhibitor rescues MFG-E8 expression in LPS-challenged RAW264.7 macrophage-like cells. **(A)** Uptake of SNA-NCs by RAW264.7 cells. The cells (n = 3 per group) were treated with SNA-NC (10 nM). They were harvested at indicated time points followed by ICP-MS assay as described in Methods. **(B)** Strategy for synthesis of SNA-NC^anti-miR99b^. Citrate-coated AuNPs were functionalized with monolayer of anti-miR99b (single-stranded antisense oligonucleotides against miRNA-99b) via thiol-gold bond and sequential addition of NaCl. **(C)** SNA-NC-conjugated anti-miRNA-99b oligonucleotides induced MFG-E8 gene expression in RAW264.7 cells. The cells were treated with or without LPS (100 ng/ml), SNA-NC^anti-miR99b^ (10 nM) and SNA-NC^scramble^ (10 nM) as indicated for 18 h followed by western blotting to measure MFG-E8 expression.

It has been shown that spherical nucleic acid nanoparticle conjugates (SNA-NC) are effective vehicles for the delivery of small oligonucleotides *in vitro* and *in vivo*^21^,^22^,^23^. Thus, we tested the hypothesis that miR-99b mediates the LPS effect on repression of MFG-E8 gene expression using SNA-NC-based approach. First, we examined the uptake of SNA-NC by macrophage-like cells. To this end, RAW264.7 cell gold content was measured following treatment with SNA-NC. As shown in Fig. 4A, uptake of SNA-NC by RAW264.7 cells occurred within 6 h in a time dependent manner, suggesting that it is feasible to deliver small oligonucleotides into macrophages using the SNA-NC-based nanotechnology. Thereafter, we synthesized a SNA-NC that was conjugated with oligonucleotides of miR-99b inhibitor and named it as SNA-NC^anti-miR99b^ (Fig. 4B). Treatment with SNA-NC^anti-miR99b^ resulted in marked increase in MFG-E8 expression in not only naïve but also LPS-challenged RAW264.7 cells (Fig. 4C). In addition, SNA-NC^anti-miR99b^ does not affect TNF-α production in LPS-challenged RAW264.7 cells (Fig. S1). Taken together, the data suggest that SNA-mediated delivery of miRNA-99b inhibitor specifically targets miR-99b-regulated MFG-E8 gene expression in RAW264.7 cells in physiological and inflammatory conditions.

**Figure 5:**
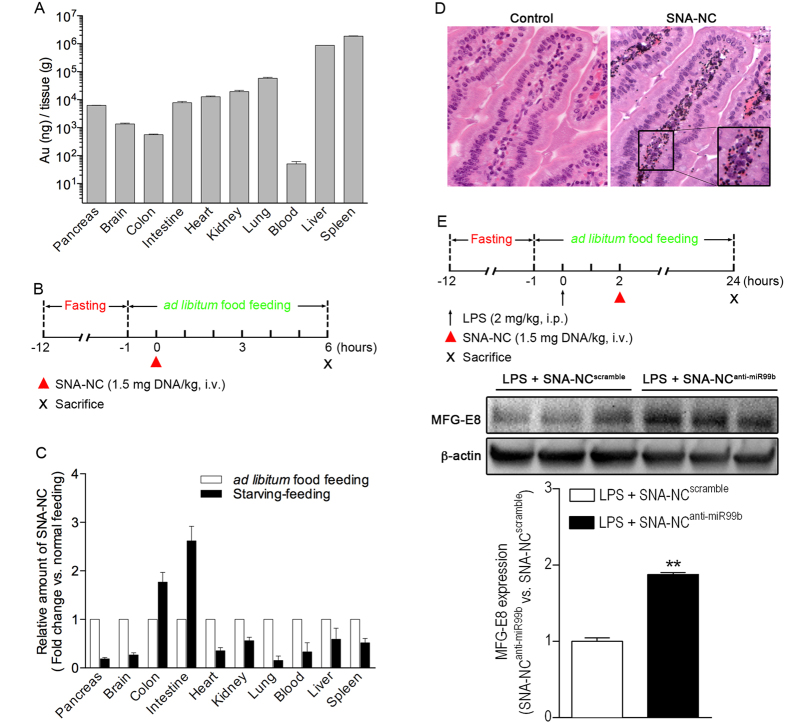
SNA-mediated delivery of miRNA-99b inhibitor enhances intestinal MFG-E8 protein expression in LPS-induced septic mice. **(A)** Tissue distribution of SNA-NC. C57BL/6 J mice (male, 7 weeks old) were intravenously injected with SNA-NC (1.5 mg DNA/kg). After 6 h, mice were sacrificed with CO_2_ inhalation and organs were processed for measuring AuNP content by ICP-MS as described in Methods. Results are reported as AuNP ng/gm tissue. Data are expressed as means ± SEM of 2 determinations. n = 2 animals/group. **(B)** Schematic diagram of starving-feeding strategy for delivery of SNA-NC. **(C)** Improving accumulation of SNA-NC in murine intestines by starving-feeding strategy. C57BL/6 J mice (male, 7 weeks old) were subjected to starving-feeding protocol and SNA-NC (1.5 mg DNA/kg, i.v.) treatment as described in Fig. 5B. After 6 h, mice were sacrificed with CO_2_ inhalation and organs stated in the figure were processed for measuring AuNP content by ICP-MS as described in Methods. Results are reported as AuNP ng/gm tissue. Data are expressed as means ± SEM of 2 determinations. n = 2 animals/group. **(D)** SNA-NC is localized in intestinal lamina propria. Intestinal tissues obtained from the experiment stated in Fig. 5C were processed for silver staining followed by microscopic examination. 40X and 63X (insert). **(E)** Treatment with SNA-NC^anti-miR99b^ results in increase in MFG-E8 expression in septic mice. Mice (male, 7 weeks old) were subjected to starving-feeding and treatment with LPS (2 mg/kg, i.p.) followed by administration of SNA-NC^anti-miR99b^ (1.5 mg DNA/kg, i.v.) using a protocol as described in the top panel. LPS-challenged mice in the control group were treated with SNA-NC^scramble^ instead. At the end of the experiment, mice were sacrificed with CO_2_ inhalation and intestinal tissues were processed for protein extraction followed by western blotting to measure MFG-E8 protein (middle panel) and densitometric analysis of immunoblot data (bottom panel). **P < 0.01 vs. SNA-NC^scramble^. Data are expressed as means ± SEM of 2 determinations. n = 3 mice/group.

Next, we examined whether administration of SNA-NC^anti-miR99b^ affected MFG-E8 expression in the small intestine. In a pilot study, a broad tissue distribution of SNA-NC was observed 6 h after intravenous (i.v.) administration (Fig. 5A). In addition, we developed a starving-feeding strategy (Fig. 5B) that showed the effect of improving the delivery of SNA-NC to the intestinal tissue (Fig. 5C). Histology examination demonstrated that a large amount of intravenously administered SNA-NC was present in intestinal lamina propria (Fig. 5D), suggesting that SNA-NC is able to facilitating delivery of small nucleotides to macrophages in the gut. Furthermore, we examined the activity of SNA-NC^anti-miR99b^ on MFG-E8 gene expression in septic mice because prior work has demonstrated that sepsis is associated with decrease in MFG-E8 gene expression *in vivo*^6^,^24^. To this end, mice were subjected to treatment with LPS (2 mg/kg, intraperitoneal (i.p.)) plus SNA-NC^anti-miR99b^ (1.5 mg DNA/kg, i.v.) using a protocol outlined in Fig. 5E, top panel. The dose of SNA-NC was chosen based on previous *in vitro* and *in vivo* gene regulation studies involving SNAs^25^,^26^. The mice in the control group were treated with LPS (2 mg/kg, i.p.) plus SNA-NC^scramble^ (1.5 mg DNA/kg, i.v.) instead. Western blotting showed that SNA-NC^anti-miR99b^ increased intestinal MFG-E8 expression in septic mice compared to SNA-NC^scramble^ treatment (Fig. 5E, middle and bottom panels). In contrast, treatment of naïve mice with SNA-NC^anti-miR99b^ alone showed no effect on MFG-E8 gene expression in the small intestine (Fig. S2). Together, the data indicate that blocking miR-99b rescued intestinal MFG-E8 expression in inflammation.

### Administration of SNA-NC^anti-miR99b^ attenuates LPS effect on inhibition of intestinal epithelial cell migration along the crypt-villus axis

**Figure 6:**
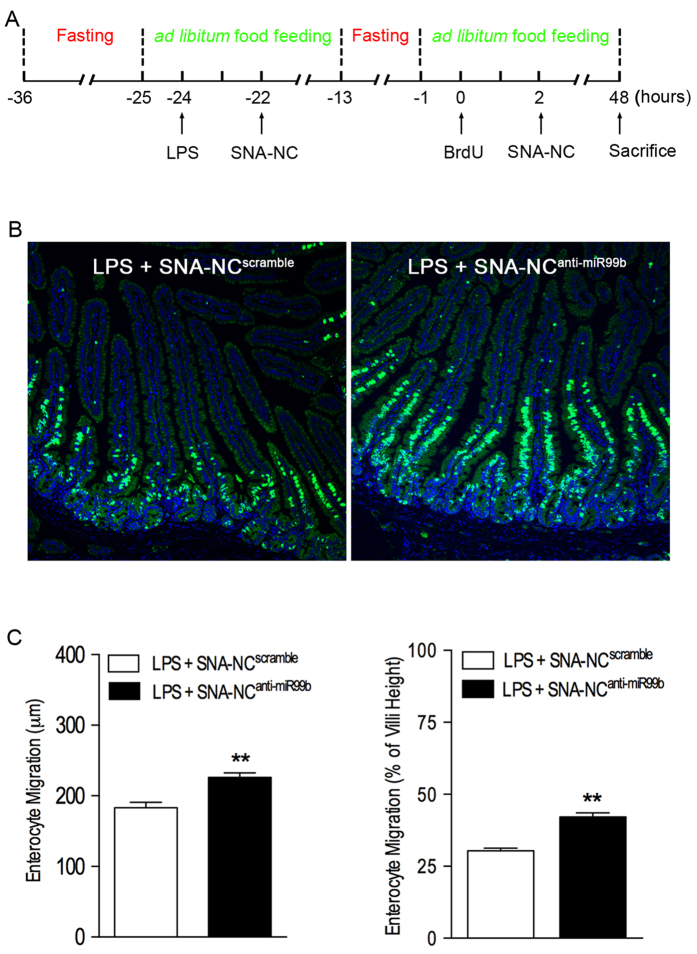
Inhibition of miR-99b promotes intestinal epithelial cells migration in LPS-induced septic mice. **(A)** Experimental design. Starving-feeding, LPS challenge (2 mg/kg, i.p.), SNA-NC treatment (1.5 mg DNA/kg, i.v.), and BrdU injection (50 mg/kg, i.p.) were executed as indicated in the figure. At the end of the experiment, mice were sacrificed with CO_2_ inhalation and the small intestine was processed for routine histology. Tissue sections were stained with anti-BrdU antibody using immunofluorescent technique. Nuclei were counterstained with DAPI. BrdU-labeled enterocyte migration was analyzed as described in Methods. **(B)** Typical photographs of the small intestine processed for BrdU/DAPI staining are shown. Merged images. BrdU-labeled cells are colored green. Nuclei are colored blue. 10X. **(C)** Quantitative analysis revealed that SNA-NC^anti-miR99b^ treatment promoted enterocyte migration along the crypt-villus axis in LPS-induced septic mice. n = 3. **P < 0.01 versus SNA-NC^scramble^ group.

In this experiment, we tested the hypothesis that SNA-NC^anti-miR99b^ treatment enhances enterocyte migration along the crypt-villus axis in septic mice using a protocol outlined in Fig. 6A. Briefly, mice were given LPS to induce sepsis. Then, the septic mice were treated with SNA-NC^anti-miR99b^ and enterocyte migration along the crypt-villus axis was measured using BrdU pulse-chase analysis. By measuring the distance between crypts and foremost-labeled enterocytes in villi, we found that SNA-NC^anti-miR99b^ treatment significantly promoted the migration of intestinal epithelial cells along the crypt-villus axis in septic mice (Fig. 6B,C), suggesting that targeting miR-99b rescued intestinal epithelial cell migration in septic condition. Conversely, treatment with SNA-NC^anti-miR99b^ alone has no effect on MFG-E8 gene expression in the small intestine (Fig. S2). Thus, we did not further examine whether SNA-NC^anti-miR99b^ treatment alone affects intestinal epithelial cell migration in naïve mice.

## Discussion

Intestinal MFG-E8 is derived from macrophages in lamina propria and plays an important role in maintaining intestinal epithelial cell homeostasis such as migration and restitution^6^,^14^,^15^,^16^. Previously, we reported that polymicrobial sepsis induced by cecal ligation and puncture (CLP) causes impairment of intestinal epithelial cell migration via down-regulation of MFG-E8 expression in intestines^6^. Furthermore, we and others have shown that LPS directly inhibits MFG-E8 gene expression in macrophages^18^,^27^. In the present study, we demonstrated for the first time that LPS-induced sepsis impairs intestinal epithelial cell migration along the crypt-villus axis. This distinctive pathophysiological feature is similar to that in CLP-induced polymicrobial sepsis^6^. Furthermore, we showed that enhancing MFG-E8 gene expression via targeting miRNA-99b (an inhibitory miRNA for MFG-E8) preserves intestinal epithelial cell migration. Taken together, our findings suggest that MFG-E8 and its regulators could be novel therapeutic targets for maintaining intestinal epithelial homeostasis in sepsis.

Evidence suggests that critical illness and severe inflammation are associated with down-regulation of intestinal MFG-E8 contents, which subsequently contributes to impairment of intestinal epithelial barrier function^6^,^17^. Thus, it is important to understand how inflammation influences MFG-E8 gene expression. Previously, we characterized the promoter region of the mouse MFG-E8 gene and demonstrated that the interaction among transcription factors Sp1, c-Jun, and their binding motifs in the promoter region plays a critical role in transcriptional regulation of the MFG-E8 gene expression in inflammation^18^. However, it remains unclear whether MFG-E8 gene expression is also regulated post-transcriptionally in inflammation. It has been shown that the interaction between 3′UTR and miRNA influences regulation of gene expression post-transcriptionally^19^,^20^. In the present study, we further examined whether and how 3′UTR of MFG-E8 mRNA is involved in regulating MFG-E8 gene expression in inflammation. We showed that MFG-E8 3′UTR controls the gene expression. It harbors a large amount of microRNA-binding sites, suggesting that MFG-E8 3′UTR can be targeted by miRNAs. We revealed that LPS induces miR-99b expression in macrophages. Furthermore, we demonstrated that miR-99b directly suppresses MFG-E8 3′UTR activity, which in turn results in down-regulation of MFG-E8 gene expression in inflammation. The data together with our previous findings suggest that inflammation affects MFG-E8 gene expression through several mechanisms including the promoter-transcription factor interaction that affects the gene expression at the transcriptional level and miR-99b-associated pathway that targets the gene expression at the post-transcriptional level.

miR-99b is a member of the miR-99b/let-7e/miR-125a cluster. Evidence shows that miR-99b targets the expression of molecules in signaling pathways associated with cell proliferation and wound healing through binding sites in their 3′UTRs. For example, previous studies demonstrated that miR-99b suppresses IGF-1R expression and contributes to inhibition of cell proliferation^28^,^29^. In addition, miR-99 family has been shown to regulate AKT/mTOR signaling by targeting mTOR and AKT1, which may play an important role in wound healing^30^. In the present study, we found that miRNA-99b directly suppresses expression of MFG-E8, a macrophage-derived potent intestinal epithelial cell restitution factor. Furthermore, we showed that targeting miRNA-99b preserves intestinal epithelial cell restitution in inflammation. Together, our data in combination with previous findings suggest that miRNA-99b is a potential therapeutic target for intestinal epithelial wound healing.

Antagomirs are a class of chemically engineered single-stranded oligonucleotides that possess activity of anti-miRs and regulate gene expression^31^. Inhibition of miRNAs using antagomirs is a novel therapeutic approach for human diseases^32^,^33^. In general, *in vivo* administrated free antagomirs often rapidly accumulate to the liver. Thus, their efficacy in targeting gene expression in solid organs other than liver may be limited without using a vehicle for delivery. To overcome this barrier, several approaches have been applied to improve delivery of small nucleotides to residential cells in solid organs. For example, coating nucleic acids with targeting moieties is shown to enhance delivery efficacy^34^. In addition, nucleic acids can be functionalized with polymers like polyethylene glycol (PEG) to increase circulation time in systemic administration^35^. Targeting moieties on nucleic acids may improve selective uptake of the molecules by residential cells in tissues over cells in blood.

Recently, we reported that the SNA-NC construct is a novel carrier that comprises a 3-dimentional (3D) core surrounded by a dense, highly orientated shell of covalently immobilized nucleic acids such as siRNA inhibitors^36^. This 3D construct is a high efficient vehicle for delivering small oligonucleotides *in vivo*. It permits intracellular delivery of large amounts of single-stranded oligonucleotides such as antagomirs for gene regulation and therapy through a single-entity of cells. Therefore, the effective dose of antagomirs carried by SNA-NC constructs is expected to be lower than that administrated by treatment with free antagomirs due to high delivery efficiency of SNA-NC. We previously found that SNA-NCs display the high ability to effectively deliver single-stranded oligonucleotides to cells^21^,^37^. It appears that intracellular release of antagomirs from engulfed SNA-NCs does not require any cellular machinery. Antagomirs delivered by SNA-NCs can function as a molecular sponge that directly binds to complementary miRNAs. We have reported that cellular internalization of SNA-NCs is dependent on caveolin-mediated endocytosis and the presence of the class A scavenger receptors^26^. Macrophages express high levels of macrophage scavenger receptor-1^38^. Thus, there is a strong rationale for delivery of antagomirs to macrophages using SNA-NC as a vehicle. In the present study, macrophages were found to engulf SNA-NCs. We demonstrated that SNA-NC^anti-miR99b^ rescued MFG-E8 expression in macrophages under both steady state and LPS-induced inflammatory conditions. To our knowledge, this is the first time that SNA-NC technology has been demonstrated to be suitable for *in vivo* targeting gene expression in macrophages. In addition, our data further support the notion that miR-99b directly regulates MFG-E8 expression.

Previously, we applied the SNA-NC system as topical forms of gene therapy for skin diseases^39^. In the present study, we further showed that a single dose of SNA-NC^anti-miR99b^ (1.5 mg DNA/kg) was sufficient to achieve a significant therapeutic response in rescuing intestinal MFG-E8 expression. Furthermore, treatment with SNA-NC^anti-miR99b^ (1.5 mg DNA/kg) showed potency on rescuing intestinal epithelial cell migration along the crypt-villus axis in septic mice. In contrast, previously studies suggested that the effective dose of carrier-free antagomirs ranged from 80 – 120 mg nucleic acid/kg *in vivo*^40^. Thus, our work represents a novel effective delivery system for targeting miRNA-regulated gene expression in intestines.

Currently, effective systemic delivery of anti-microRNA to a solid organ other than liver or lung for fulfilling a therapeutic purpose remains a challenge^41^. In the present study, we applied a typical feeding strategy in order to improve intestinal distribution of SNA-NC^anti-miR99b^. Briefly, this approach contains two steps including fasting overnight and feeding for one hour prior i.v. administration of SNA-NC^anti-miR99b^. We demonstrated that the absolute bio-distribution of SNA-NC in intestines increased 2.62-fold (±0.30, *P* < 0.05) when compared to mice subjected to a standard feeding strategy before injection of SNA-NC^anti-miR99b^ (Fig. 5C). This effect is most likely due to increase in intestinal blood flow following rapid food intake after starvation^42^.

Finally, administration of LPS to experimental animals produces a number of systemic effects similar to gram-negative sepsis such as systemic arterial hypotension (i.e. shock), lactic acidosis, impaired myocardial contractility, and alteration of metabolic profiles. On the other hand, LPS-induced sepsis has been shown to be different from CLP-induced sepsis in numerous respects, especially regarding the kinetics and magnitude of cytokine production^43^. In the present study, we demonstrated that injecting mice with LPS via the intraperitoneal route leads to impairment of intestinal epithelial cell migration *in vivo*, which are reminiscent of the pathophysiology of intestinal barrier dysfunction in CLP-induced polymicrobial sepsis^6^. Thus, impairment of intestinal epithelial homeostasis seems to be a common gut-associated pathophysiological feature in sepsis and severe systemic inflammation.

**Figure 7:**
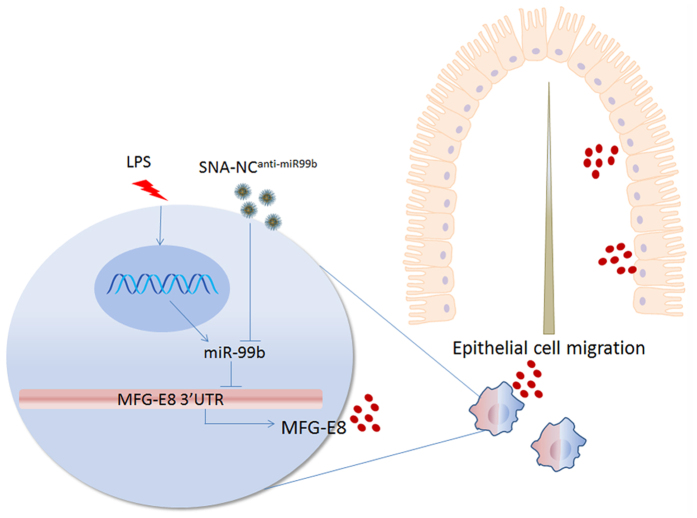
Working model for the effect of targeting MFG-E8 gene expression in macrophages on intestinal epithelial homeostasis. The septic challenges such as severe endotoxemia induce miR-99b levels, which subsequently inhibits expression of MFG-E8 at post-transcriptional levels via targeting 3′UTR of MFG-E8 mRNA in intestinal macrophages. Down-regulation of MFG-E8 expression contributes to impairment of intestinal epithelial cell migration along the crypt-villus axis in sepsis. Administration of SNA-NC^anti-miR99b^ neutralizes miR-99b in macrophages. This results in rescued MFG-E8 expression, with the resultant attenuation of the effect of sepsis on inhibiting intestinal epithelial cell migration *in vivo*.

In summary, we show that LPS inhibits MFG-E8 expression through induction of miR-99b that targets 3′UTR of MFG-E8 mRNA. LPS-induced down-regulation of MFG-E8 is associated with impairment of intestinal epithelial cell migration along the crypt-villus axis. We established a strategy to deliver SNA-NC to intestines. We further demonstrated that antagomir against miR-99b carried by SNA-NC rescues MFG-E8 expression and intestinal epithelial cell restitution in inflammation. In addition, we previously demonstrated that treatment with MFG-E8 directly rescues intestinal epithelial cells migration along the crypt-villus axis in septic mice^6^. Collectively, our studies suggest the notion that miR-99b and MFG-E8 are potential targets for sustaining intestinal epithelial barrier integrity in inflammation (Fig. 7).

## Material and Methods

### Cell culture

RAW264.7 cells (a mouse macrophage-like cell line, passages 10–18 after receipt from American Type Culture Collection) were cultured in a water-saturated atmosphere with 5% CO_2_ at 37 °C in Dulbecco’s modified Eagle’s minimum essential medium supplemented with 10% heat-inactivated fetal bovine serum, 1% nonessential amino acids, 100 U/ml penicillin, and 100 μg/ml streptomycin.

### RNA extraction and quantitative real-time RT-qPCR

RNA was isolated from 1 × 10^6^ RAW264.7 cells using the RNeasy RNA extraction kit purchased from QIAGEN (Hilden, Germany) and reverse-transcribed to single-stranded cDNA using the iScript™ cDNA synthesis kit obtained from Bio-Rad laboratories (Hercules, CA) according to the manufacturer’s protocols. Quantitative real-time PCR for measuring MFG-E8 gene transcripts was performed using a standard protocol as previously described^15^,^44^. Sequences of forward (F) and reverse (R) primers for real-time PCR were murine MFG-E8-F 5′-ATCTACTGCCTCTGCCCTGA-3′, murine MFG-E8-R 5′-CCAGACATTTGGCATCATTG-3′, murine 18S rRNA-F 5′-TGCCCTATCAACTTTCGATG-3′, and murine 18S rRNA-R 5′-GATGTGGTAGCCGTTTCTCA-3′. The expression levels of MFG-E8 mRNA in samples were calculated using the 2^−ΔΔ*C*T^ method. 18S rRNA served as a control gene for calculating the Δ*C*_*T*_(Δ*C*_*T*_ = *C*_*T*mfge8_ − *C*_*T*18S_). The ΔΔ*C*_T_ value is defined as the CT difference between the normalized amount of sample and the normalized amount of calibrator.

### Protein extraction and Western blotting

Total cellular proteins were isolated from cells or intestinal tissues using a protocol as previously described^44^. Protein extracts (15 μg) were fractionated in NuPAGE^®^ 4–12% Bis-Tris Gels (precast polyacrylamide gels supplied by Invitrogen) followed by electrophoretically transferred to PVDF membranes. Goat polyclonal antibody against murine MFG-E8 (1:800, R&D Systems) was used to detect MFG-E8 protein in blots. Horseradish peroxidase (HRP)-conjugated anti-goat IgG polyclonal antibody (1:3,000, Life Technologies) was used as the secondary antibody. After washing with PBS-T, membranes were treated with a development solution supplied in ECL plus kit (Thermo Scientific, Rockford, IL), scanned with ChemiDoc^TM^ imaging system (Bio-Rad, Hercules, CA), and analyzed with Imaging Lab 4.1 software (Bio-Rad, Hercules, CA). For detection of house-keeping gene expression, blots were stripped and re-probed with an HRP-conjugated mouse mAb against β-actin (1:50,000, clone AC-15, Sigma-Aldrich, St. Louis, MO) followed by development with ECL plus kit, scanning and analyzing as described above.

### microRNA array and microRNA validation

Total RNAs including microRNAs were extracted from cells using miRNeasy mini kit (QIAGEN). RNA samples were processed for microRNA array by EXIQON (Woburn, MA). MicroRNA candidates targeting on MFG-E8 3′UTR were predicted using a database available at http://microrna.sanger.ac.uk/targets/v5/ followed by validating their expression profiles using the TaqMan RT-qPCR kit (Life Technologies, Grand Island, NY).

### Preparation of MFG-E8 3′UTR luciferase reporter construct

MFG-E8 3′UTR luciferase reporter construct was generated by Switchgear Genomics (Merlo Park, CA). Briefly, the DNA fragment corresponding to the mouse MFG-E8 3′UTR was inserted into Nhe-XhoI cloning sites of a luciferase miRNA Target Expression Vector, namely, pSGG (Switchgear Genomics, CA). The inserted MFG-E8 3′UTR fragment in the pSGG vector was confirmed by sequencing. The new plasmid was named as pSGG^mfge8-3′UTR^.

### Transfection and luciferase reporter assay

RAW264.7 cells were plated onto 24-well plates at 4 × 10^4^ cells per well. After 24 h, cells in each well were transfected with a plasmid mixture containing 0.1 μg *of* pRL-null plasmid (Promega Corp., Madison, WI) and 0.3 μg of either pSGG^mfge8-3′UTR^ *using* Lipofectamine 2000 transfection reagent (Life Technologies, Grand Island, NY). The control cells were transfected with a mixture of pRL-null plasmid and *empty pSSG vector instead*. The manufacturer’s protocol was followed for running transfection reactions. Forty-eight hours after transfections, cells were processed for measuring luciferase activity using Dual-Luciferase Reporter Assay System (Promega Corp.) using a protocol provided by the manufacturer. Wallac Victor^2^ 1420 Multilabel Counter (PerkinElmer, Waltham, MA) was used for measuring levels of bioluminescence generated in the assay. Levels of the firefly luciferase activity represented the activity of pSGG^mfge8-3′UTR^ or pSGG empty vector, whereas the Renilla luciferase activity was used for normalization of the transfection efficiency. For each transfection, luciferase activity was averaged from six replicates.

### Preparation of SNA-NC containing anti-miR99b antisense oligonucleotide

The antisense oligonucleotides against miR-99b (i.e. miR-99b inhibitor) were synthesized on an MM48 Oligonucleotide Synthesizer (BioAutomation, Irving, TX) using standard solid-phase synthesis and reagents (Glen Research). All DNAs were purified using reverse-phase high performance liquid chromatography (Varian system, Aglient Technologies, CA) with a Microsorb^TM^ C18 column (Varian, Inc. CA). Sequence of the miR-99b inhibitor is: 5′-CGCAAGGTCGGTTCTACGGGTG(A_10_)-3′-SH. To functionalize the gold nanoparticles, propylthiol functionalized DNA was added to 10 nM citrate-capped gold nanoparticles (AuNPs, 13 nm in diameter) at a concentration of 1 OD of DNA per mL of 10 nM AuNPs supplemented with 0.1% Tween 20. After stirring at room temperature for 1 h, the solution was aged by the gradual addition of NaCl over 6 h to bring the final NaCl concentration to 0.5 M. Functionalized AuNPs were separated from free DNA strands via dialysis against Nanopure water using a 50-kDa Amicon molecular weight cutoff (MWCO) membrane (Millipore, MA). AuNP and DNA concentrations were determined by measuring their extinction at 524 nm and 260 nm, respectively, on a Cary 5000 ultraviolet/visible (UV-Vis) spectrophotometer (Aglient Technologies, CA). The SNA-NC product containing miR-99b inhibitor was denoted as SNA-NC^anti-miR99b^.

### Quantification of SNA cellular uptake and tissue distribution using inductively coupled plasma mass spectroscopy (ICP-MS)

Samples from cells and mouse tissues were harvested for ICP-MS to measure gold content which quantitatively represents SNA cellular uptake and biodistribution *in vivo*. To determine the cellular uptake kinetics of SNA-NCs in mouse macrophages, RAW264.7 cells were seeded in a 24-well plate at a population of 5 × 10^4^ cells per well 12 h in advance of particle treatment at 37 °C and 5% CO_2_. Cells were incubated with 0.3 ml of serum reduced OptiMEM culture medium containing 10 nM SNA-NCs per well. Samples were harvested at 0, 10, 20, 30, 60, 120 and 240 min of treatment (n = 3 for each time point) after rinsing with OptiMEM and trypsinization for counting using a hemacytometer and centrifugation at 8,000 rpm for 5 min to form a pellet for quantification by ICP-MS. The cell pellets were digested with 0.3 ml of aqua regia (concentrated HCl : concentrated HNO3, 3:1, v/v,) with trace metal at room temperature overnight. For *in vivo* experiments, tissue samples collected after mice dissections were dried, weighted, and digested with 1 ml of aqua regia in a 50 °C oven overnight. After adding 5 μl of 5 ppm indium (internal standard) and 5 ml of matrix solution (2% HCl and 2% HNO_3_), the Au-197 content of the resultant solution was measured by an X Series II ICP-MS (Thermo Fisher) after subtracting the background gold content of untreated cells. Unless otherwise mentioned, reported values represent mean ± SEM from the average of three independent experiments^45^.

### Animals

C57BL/6 J mice (male, specific pathogen-free 7 weeks old) were purchased from the Jackson Laboratory (Bar Harbor, ME). They were housed in the specific pathogen-free animal facility at Stanley Manne Children’s Research Institute. All animal experiments were conducted according to the experimental procedures approved by the Institutional Animal Care and Use Committee of Stanley Manne Children’s Research Institute.

### LPS treatment and analysis of enterocyte migration *in vivo* with the BrdU labeling strategy

The traditional endotoxicosis model of sepsis was used to determine the effect of sepsis-associated overwhelming innate immune response on intestinal epithelial cell migration *in vivo*^43^,^46^. Briefly, mice were injected intraperitoneally with LPS (2 mg/kg; serotype 0111:B4; Sigma, St Louis, MO) in normal saline. For labeling intestinal epithelial cells in crypts, mice were intraperitoneally injected with BrdU (50 mg/kg, BD Biosciences) at 24 h after LPS treatment. Mice were sacrificed with CO_2_ inhalation at 48 h after BrdU administration. The entire small intestine was removed, flushed with cold saline, fixed with 10% buffered formalin, and processed for paraffin embedding and sectioning. Sections (5 μm) were processed for BrdU staining and counterstaining with 4′,6-diamidino-2-phenylindole (DAPI) using our standard protocol^6^. Then, BrdU-labeled cells were visualized and analyzed as described before^6^.

### Protocol for enhancement of delivery of SNA-NC to the gut in mice

Briefly, mice were fasted overnight then fed with food the next morning. One hour after feeding, mice were i.v. injected with SNA-NC (1.5 mg DNA/kg). They were sacrificed with CO_2_ inhalation at adequate time points after administration of SNA-NCs. Then, the small intestine was harvested and flashed with cold saline. A portion of intestinal tissues was fixed in 10% buffered formalin and processed for routine histological examination, whereas others were processed for molecular biological studies.

### Silver-staining

Silver staining was performed by using the Silver Enhancing kit (BB International, UK) using the protocol suggested by Northwestern University histological core facility.

### Statistical analysis

Data were subjected to analysis by either the student’s t-test or ANOVA followed by Fisher’s least significant difference post-hoc test. All experiments were performed at least twice. Data are expressed as means ± SEM. *P* < 0.05 was considered significant.

## Additional Information

**How to cite this article**: Wang, X. *et al*. Spherical nucleic acid targeting microRNA-99b enhances intestinal MFG-E8 gene expression and restores enterocyte migration in lipopolysaccharide-induced septic mice. *Sci. Rep.* **6**, 31687; doi: 10.1038/srep31687 (2016).

## Supplementary Material

Supplementary Information
