# Supplementary material for: Spherical nucleic acid targeting microRNA-99b enhances intestinal MFG-E8 gene expression and restores enterocyte migration in lipopolysaccharide-induced septic mice
Source: Sci Rep. 2016 Aug 19;6:31687. doi: 10.1038/srep31687 (PMC4990839; doi:10.1038/srep31687)
Supplement: Supplementary Information [file srep31687-s1.pdf]

Spherical nucleic acid targeting microRNA-99b enhances intestinal MFG-E8 gene expression and restores enterocyte migration in lipopolysaccharide-induced septic mice

Xiao Wang, Liangliang Hao, Heng-Fu Bu, Alexander W. Scott, Ke Tian, Fangyi Liu, Isabelle G. De Plaen, Yulan Liu, Chad A. Mirkin, and Xiao-Di Tan

SUPPLEMENTARY DATA

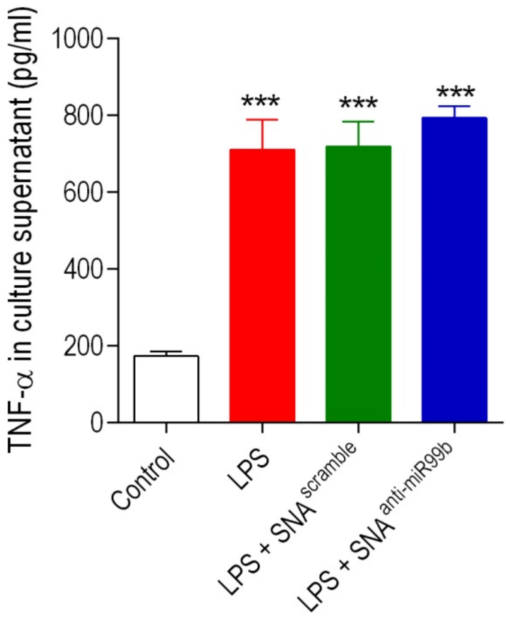

Figure S1. SNA-NC$^{anti\text{-}miR99b}$ does not affect TNF-α production in LPS-challenged RAW 264.7 macrophage-like cells.

RAW 264.7 cells were subjected to treatments with medium alone (Control), LPS (100 ng/ml), LPS + SNA-NC^{scramble} (10 nM), and LPS + SNA-NC$^{anti\text{-}miR99b}$ (10 nM) for 24 h respectively. At the end of the treatments, cell culture supernatants were harvested for measurement of TNF-α contents with an ELISA kit purchased from R & D using a protocol provided by the manufacture. n = 4. Results are the means ± SEM. ***, $P < 0.001$ compared with the control group.

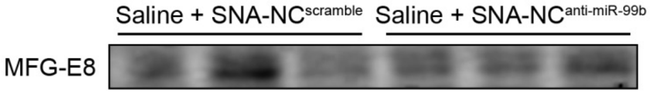

Figure S2

Figure S2. Treatment with SNA-NC$^{anti-miR99b}$ does not affect intestinal MFG-E8 expression in naïve mice.

Mice (male, 7 weeks old) were fasted for 11 h followed by ad libitum food intake. Then, they were treated with saline (200 μl/mouse, i.p.) at 1 h after initiating of food feeding and subjected to SNA-NC treatment (1.5 mg DNA/kg, i.v.) at 3 h after initiating of food feeding. At 23 h after saline treatment, mice were sacrificed with CO_2 inhalation. Intestinal tissues were processed for protein extraction followed by western blotting to measure MFG-E8 protein.
